# Application of multi-criteria decision analysis techniques and decision support framework for informing plant select agent designation and decision making

**DOI:** 10.3389/fbioe.2023.1234238

**Published:** 2023-09-11

**Authors:** Segaran P. Pillai, Julia Fruetel, Todd West, Kevin Anderson, Patricia Hernandez, Cameron Ball, Carrie McNeil, Nataly Beck, Stephen A. Morse

**Affiliations:** ^1^ Office of the Commissioner, Food and Drug Administration, Silver Spring, MD, United States; ^2^ U.S. Department of Health and Human Services, Washington, DC, United States; ^3^ Sandia National Laboratories, U.S. Department of Energy, Livermore, CA, United States; ^4^ Retired, Livermore, CA, United States; ^5^ Retired, Palmetto, FL, United States; ^6^ Retired, Atlanta, GA, United States

**Keywords:** multi-criteria decision analysis (MCDA), decision support framework (DSF), plant select agents, biennial review, risk assessment tool

## Abstract

The United States Department of Agriculture (USDA) Division of Agricultural Select Agents and Toxins (DASAT) established a list of biological agents (Select Agents List) that threaten crops of economic importance to the United States and regulates the procedures governing containment, incident response, and the security of entities working with them. Every 2 years the USDA DASAT reviews their select agent list, utilizing assessments by subject matter experts (SMEs) to rank the agents. We explored the applicability of multi-criteria decision analysis (MCDA) techniques and a decision support framework (DSF) to support the USDA DASAT biennial review process. The evaluation includes both current and non-select agents to provide a robust assessment. We initially conducted a literature review of 16 pathogens against 9 criteria for assessing plant health and bioterrorism risk and documented the findings to support this analysis. Technical review of published data and associated scoring recommendations by pathogen-specific SMEs was found to be critical for ensuring accuracy. Scoring criteria were adopted to ensure consistency. The MCDA supported the expectation that select agents would rank high on the relative risk scale when considering the agricultural consequences of a bioterrorism attack; however, application of analytical thresholds as a basis for designating select agents led to some exceptions to current designations. A second analytical approach used agent-specific data to designate key criteria in a DSF logic tree format to identify pathogens of low concern that can be ruled out for further consideration as select agents. Both the MCDA and DSF approaches arrived at similar conclusions, suggesting the value of employing the two analytical approaches to add robustness for decision making.

## Introduction

More than 50,000 plant diseases have been recognized in the United States (U. S.) and there are many more that occur globally. (Nutter and Madden, 2005). Plant pathologists estimate that the majority of plant diseases are caused by fungal and oomycete pathogens (Strange, 1993; Nutter and Madden, 2005; Fletcher et al., 2011). Each year, plant diseases cost the global economy more than $220 billion and crop production loss due to pests is between 20% and 40% (NIFA, 2023).

Fletcher et al. (2011) distinguished three forms of intentional use of pathogens to infect crops: 1) biowarfare, a state-sponsored and funded activity to reduce a nation’s food resources, which includes commercial or economic sabotage for trade advantage; 2) bioterrorism, which involves small groups or single individuals with a political, social, or religious agenda; and, 3) biocrimes, which are motivated by issues such as commodity price manipulation, commercial competition, revenge, or to create a dependence on a particular product. The consequences of a biological attack on the U. S. agriculture sector may be significant due to its economic importance, representing about 20% of the U. S. export market since 2000 (USDA International Markets and U. S. Trade, 2022). Additional economic consequences could occur through loss of international markets because phytosanitary restrictions on trade, which are imposed by importing countries that are free of a particular highly contagious plant disease, will ultimately affect the economy of the exporting country (Wheelis et al., 2002).

Crops as targets offer several advantages to the perpetrator(s). Agricultural crops are often described as “soft targets” because they are grown over large acreages, making continuous and effective surveillance of them nearly impossible (Nutter and Madden, 2005). One consequence of this minimal crop surveillance is a potentially long lag time between the introduction of a pathogen and its detection (Nutter and Madden, 2005). For plant pathogens with extremely high reproductive rates (R_0_), successful eradication and containment of such a newly introduced pathogen is only possible if it is detected soon after introduction (Madden and Wheelis, 2003). Thus, early detection, containment, treatment if available, and other appropriate preventive measures are of utmost importance in limiting the spread of the pathogen. Likewise, recognition and control of certain pathogens is also key to preventing their accidental or intentional introduction.

Another advantage of targeting crops is the ease with which a plant pathogen can be introduced into the U. S. For example, bioterrorists could carry small amounts of inoculum (less than a Gram) across the long borders with Mexico or Canada to infect crops (Madden and Wheelis, 2003). Humans are generally not susceptible to infection by plant pathogens meaning no special safety precautions are required to collect, culture, reproduce, store, or deliver the inoculum to its target (Nutter and Madden, 2005). Once an agricultural pathogen has been introduced to a new area, forensic attribution can be extremely difficult because mutations may accumulate during the potentially long time it may take to correctly detect and identify the pathogen (Fletcher et al., 2011).

The strategic use of biological weapons (BWs) against plants by state programs was considered as a means to cause economic damage or to reduce the enemy’s food supplies (Whitby, 2006). After World War II (WWII), the development of anti-crop BWs was pursued by programs in the U. S., United Kingdom (U.K.), Soviet Union, Iraq, and others. Research in the U. S. focused on fungal plant pathogens and other agents for use against rice, potatoes, tobacco, sugar beets, sweet potatoes, and cotton. Most of the research centered on the causative agents of stem rust of wheat (*Puccina graminis*), rice blast (*Piricularia oryzae*), and late blight of potatoes (*Phytophthora infestans*). Other anti-crop agents under review by the U. S. for their potential as BWs included *Puccinia striiformis* (stripe rust of wheat), Hoja Blanca virus (Hoja Blanca of rice), *Xanthomonas oryzae* (Uyeda et Ishiyama - bacterial leaf blight of rice), and *Peronospora arborescens* (downy mildew of poppy) (Whitby, 2006). At the time the U. S. program was terminated, its BW stockpile contained 158,684 pounds of *P. graminis* var. *tritici* and 1,865 pounds of *X. oryzae* (van Courtland Moon, 2006). Studies showed that the *P. graminis* var. *tritici* was very potent with an infectious dose of 0.1 g/acre or 1 pound/10 square miles with aerosolized spores remaining viable for several days (Whitby, 2006).

After WWII, the Soviet Union established the Ekologiya Program whose mission was to develop viruses, bacteria and fungi that would destroy animals and plants important to U. S. agriculture, including pathogens that attacked wheat, rye, potatoes, corn, and rice (Leitenberg and Zilinskas, 2012). Most of the developmental research was conducted at facilities under the Ministry of Agriculture including the Scientific Institute of Phytopathology in Tashkent, Uzbekistan where anti-crop weapons were researched and developed; the Scientific Institute of Phytopathology in Golitsino, Russia, which developed anti-crop weapons, including agents for the destruction of wheat, rye, corn, and rice; and the Scientific Institute and Test Site at the Otar Railway Station, Kazakhstan where anti-crop BWs were tested (Alibek and Handelman, 1999). However, unlike the U. S. program, the Soviet program did not stockpile anti-crop weapons, but rather relied on its capacity to rapidly produce them when needed (Leitenberg and Zilinskas, 2012).

Convincing evidence for prior use of anti-crop BWs by state programs is scant to non-existent (Carus, 2002; Whitby, 2006; Zilinskas, 1999). However, the government of Cuba alleged on several occasions that it was the victim of biological warfare operations conducted by the U. S. (Zilinskas, 1999). These allegations included the introduction of fungi responsible for tobacco blue mold disease (*Peronospora tabacina*) in 1979–80, and sugarcane rust disease (*Puccinia melanocephalo*) in 1979. However, no credible evidence was found supporting these claims and alternative natural explanations for these outbreaks were considered more likely (Zilinskas, 1999).

The deliberate misuse of biological agents posing a threat to the agricultural sector and the food chain has been termed agroterrorism (Ryan and Glarum, 2008). The threat of agroterrorism has led to the promulgation of regulations in the U. S. to ensure the biosafety and biosecurity of plant pathogens. A list of high threat pathogens for humans (select agents) has existed since 1997 (Morse, 2015); however, the addition of comparable pathogens for animals and plants did not occur until after the passage of the Public Health Security and Bioterrorism Preparedness and Response Act in 2002 (Public Law 107-188, 2002). Subtitle B (Agricultural Bioterrorism Protection Act of 2002) of PL 107–188 directed the Secretary of the USDA “to establish and maintain a list of biological agents and toxins that he/she determined have the potential to pose a severe threat to plant health or products. The criteria for inclusion on this list included: 1) the effect of an agent or toxin on plant health or products and marketability of plant products; 2) the virulence of an agent or degree of toxicity of the toxin and the methods by which the agents or toxins are transferred to plants; 3) the availability and effectiveness of treatments (e.g., fungicides) for any illness caused by an agent or toxin; and 4) other criteria that the USDA Secretary considers appropriate to protect plant health or plant products” (CFR Title 7, Subtitle B, Chapter 3 part 331). The plant Select Agent list is reviewed and re-evaluated on a biennial basis (Table 1). However, there is a significant difference between Select Agent lists for humans and animals and that for plants. While the former lists include both endemic and exotic pathogens, plant pathogens that are established (i.e., unlikely to be eradicable) in the U. S. are excluded or delisted when they enter the U.S. and become established. For example, pathogens that were on the list at one time (e.g., *Phakopsora pachyrhizi*, plum pox potyvirus, *Xylella fastidiosa*, *Liberibacter africanus*, and *Liberibacter asiaticus*) were delisted after they entered and became established in the U. S. (Table 1).

**TABLE 1:** Changes to the USDA/APHIS Plant Select Agent List since its inception.

	Year				
Pathogen	2002 (inception)	2005	2008	2012	2018
*Candidatus Liberibacter africanus*	Included		Delisted		
*Candidatus Liberibacter asiaticus*	Included		Delisted		
*Coniothyrium glycines* (formally *Phoma glycinicola* and *Pyrenochaeta glycines*)			Added		
*Peronosclerospora philippinensis (Peronosclerospora sacchari)*	Included				
*Phakopsora pachyrhizi*	Included	Delisted			
Plum pox potyvirus	Included	Delisted			
*Ralstonia solanacearum* phylotype II sequevar 1 (Race 3, biovar 2)	Included				
*Rathayibacter toxicus*			Added		
*Sclerophthora zeae (raysiae)*	Included				
*Synchytrium endobioticum*	Included				
*Xanthomonas oryzae*	Included				
*Xylella fastidiosa* (citrus variegated chlorosis strain)	Included				Delisted

Recently, we published the use of MCDA and DSF logic tree analyses to assist the CDC Division of Select Agents and Toxins (DSAT) Program’s biennial review of the HHS Select Agent and Toxin list, applying the approach broadly to include non-select agents and toxins to evaluate its robustness (Pillai et al., 2022a; Pillai et al., 2022b). A description of these methodologies, their disadvantages, advantages, and prior application has been previously summarized (Pillai et al., 2022a; Pillai et al., 2022b).

In this study we evaluated whether approaches used for HHS agents would be effective in supporting deliberations and recommendations by the Agricultural Intragovernmental Select Agents and Toxins Technical Advisory Committee (Ag ISATTAC) regarding which pathogens to include on the USDA Select Agent list. Previous efforts by the Ag ISATTAC relied solely on SME assessments. In 2018, the Ag ISATTAC sought to improve upon previous approaches. Two analytical approaches were developed and evaluated for classifying plant pathogens as USDA Select Agents: an MCDA framework and a DSF logic tree. The analytical approaches we describe herein seek to provide approaches for assessing the impact on national security, and to reduce the burden on SMEs by documenting the supporting data from peer-reviewed literature in agent fact sheets to support the process. In this study the selection of agents was determined by SMEs based on their expertise focusing on high consequence exotic pathogens as required by USDA and did not include low consequence endemic pathogens.

## Methods

### Multi-criteria decision analytical framework

**FIGURE 1:**
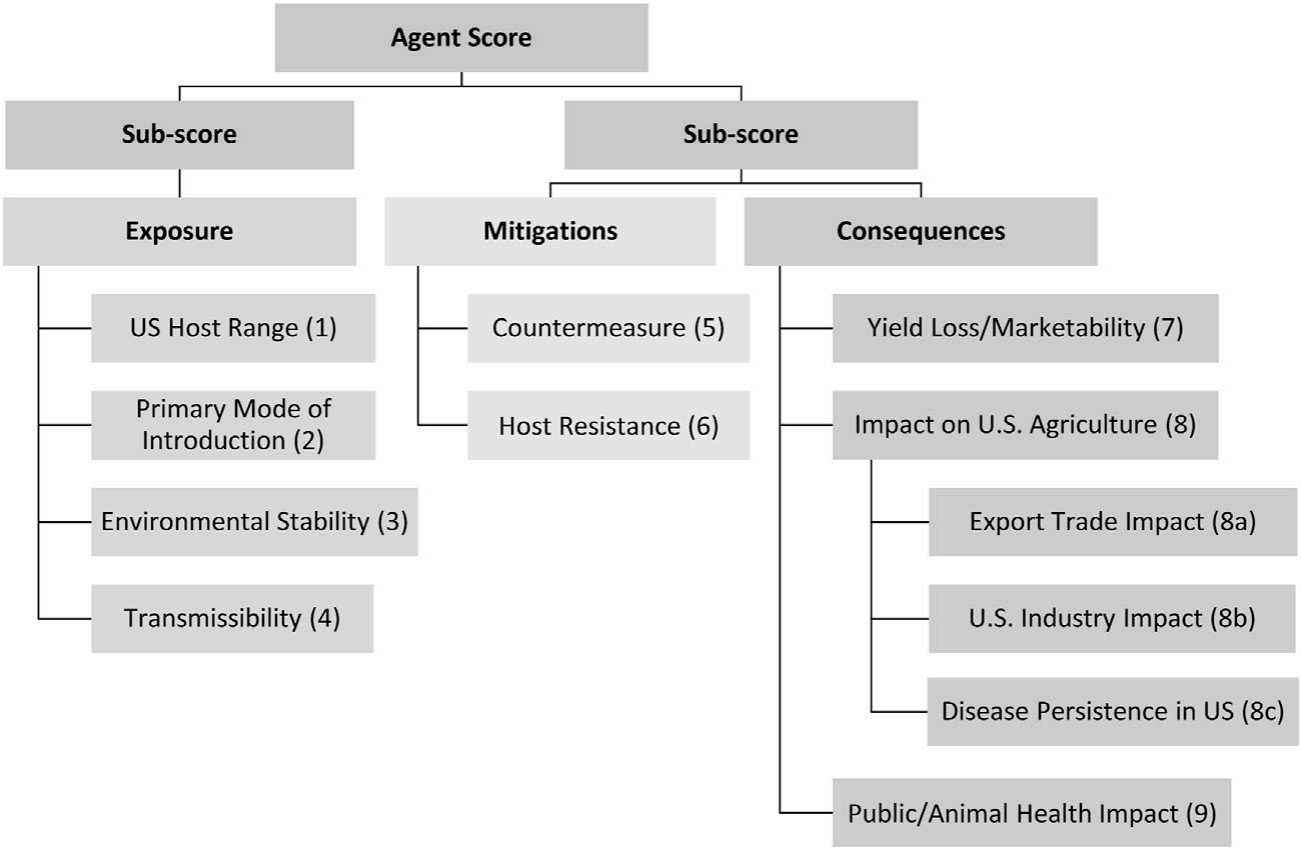
Summary of the criteria and hierarchy captured in the MCDA framework and fact sheets.

The starting point for the MCDA was a set of 9 criteria that affect bioterrorism risk assessment as set forth in Public Law 107–188, 2002. For convenience, these criteria were grouped into those relevant for agent exposure, mitigation, and consequence, which includes potential economic impact (Figure 1). Note that endemicity is not one of the nine criteria used for the MCDA. SMEs collectively scored these 9 criteria on a scale of 0–10, based on data in the agent fact sheets and using the scoring definitions in Table 2 for each of the pathogens in Table 3. The scoring system represents the level of concern applicable to the agent’s classification as a plant select agent, ranging from 0 (indicating minimal concern) to 10 (indicating maximum concern). To keep things simple, a linear scale was adopted for this assessment. During the course of the study, SME’s/authors with expertise in a particular agent or taxonomic group were asked to score the criteria and lead the discussion to achieve consensus among group members for consistency. Table 2 lists the scoring definitions for each of the criteria for even-numbered scoring options (0, 2, 4, 6, 8, and 10). Odd scores (1, 3, 5, 7, and 9) were used if an SME felt that the most appropriate score fell between the provided even score options.

**TABLE 2:** Criteria scoring definitions.

**U.S. Host Range 1)—**Hosts in the U.S. that are susceptible to the disease (e.g., corn, soybeans, wheat, citrus, *etc.*)	
0	None
2	Oats
4	Rice, sorghum, citrus or barley
6	Wheat, potatoes, forage grasses consumed by livestock, sugarcane or cotton
8	Corn or soybeans
10	Large host range (e.g., multiple U.S. crops or multiple plant families that would significantly impact the U.S. economy, i.e., vegetables, fruit and tree nuts)
**Primary Mode of Introduction 2)—**The routes in which the pathogen is introduced to susceptible hosts	
0	None
2	Vector or contaminated seed
4	Through contaminated soil or ground water
6	Through direct exposure to pathogen via aerosols
8	2 different routes
10	3 different routes
**Environmental Stability 3)—**The extent to which the pathogen is stable outside the host, in the environment (e.g., in soil, water) and on surfaces/fomites	
0	Is not stable in the environment
2	Cannot survive in the environment without a host
4	Is stable only in the absence of sunlight or moisture
6	Is stable in soil, water or as an aerosol for up to 2 years
8	Is stable in soil, water or as an aerosol for 2–9 years
10	Is stable in soil, water or as an aerosol for 10 years or more
**Transmissibility 4)**—The extent to which the disease can be transmitted from plant to plant and farm to farm	
0	None
2	Seed-borne or via nematode
4	Transmitted through fomites (e.g., utensils, tires, farm equipment, boots, diseased plant material) or via mites
6	Transmitted through vectors other than nematode and mites
8	Transmitted short distances (e.g., within a farm) by wind, movement of soil or irrigation water
10	Can be transmitted long-distance (e.g., farm to farm) by wind, water or rain
**MITIGATION**	
**Countermeasures 5)**—The availability and effectiveness of countermeasures (e.g., pesticides, fungicides, soil fumigation) and extent to which they can be rapidly deployed and administered in an emergency	
0	No countermeasures required or countermeasures already used in routine operations are effective
2	Identification and elimination of infected crop is sufficient to mitigate disease
4	Specialty countermeasures required or chemical control methods such as fungicides, fumigants and insecticides are effective and can be rapidly deployed
6	Chemical control methods (e.g., fungicides, fumigants and insecticides) are partially effective and/or cannot be rapidly deployed
8	Destructive measures such as crop tillage, burning and/or destroying infected plants and the associated soil are effective
10	No effective countermeasures exist or are feasible
**Host Resistance 6)—**The extent to which the affected U.S. crops have been genetically engineered or modified to resist disease	
0	All U.S. cultivars are resistant
2	Majority (>80%) of U.S. cultivars are resistant
4	Strains with partial resistance are available (e.g., do not protect against all pathotypes)
6	Resistant strains are available but not in common use in the U.S. (e.g., are available outside the U.S.)
8	<20% of U.S. cultivars are resistant
10	All U.S. cultivars are susceptible
**CONSEQUENCES**	
**Yield Loss/Marketability 7)—**The extent to which crop yield/marketability is lost due to disease or toxin production. Consider susceptible varieties	
0	None (or data not available)
2	<10%
4	11%–20%
6	21%–30%
8	30%–40%
10	>40%
**Impact on U.S. Agriculture 8)—**The burden to U.S. agriculture during and after an event (as measured by quarantine, export trade impacts and U.S. industry impacts)	
**Export Trade Impact (8a)—**The extent to which the crop is exported from the US as measured by percent of total US crop production in tons	
0	∼0%
2	1%–10%, or low expected impact due to existing endemic disease
4	11%–20%
6	21%–40%
8	41%–50%
10	>50%
**U.S. Industry Impact (8b)—**The size of the U.S. industry for the crops susceptible to the disease and potential impacts to the food supply beyond the expected overhead	
0	None to low impact as control measures are included in overhead costs for endemic diseases
2	<$100M, or low expected impact due to existing control measures for endemic disease
4	$100–999 M
6	$1—9B
8	$10B- 50B
10	>$50B
**Disease Persistence in U.S. (8c)—**The means by which the pathogen can persist in the U.S. following an introductory event, through harboring in vectors, reservoir populations and/or with conducive climate conditions	
0	No persistence
2	Limited persistence due to unfavorable climate conditions (temperature extremes, rainfall, *etc.*)
4	Persistence contributed by vectors
6	Persistence contributed by alternate host such as weeds and other crops
8	Moderate persistence contributed by contaminated water
10	High persistence contributed by contaminated soil
**Direct Public/Animal Health Impact 9)—**The potential impact on human or animal health from the agent	
0	Does not cause disease in humans and/or animals
2	Causes mild symptoms and/or is only rarely lethal in humans and/or animals
4	Causes moderate morbidity and low mortality (CFR <9%) in humans and/or animals
6	Causes moderate morbidity and mortality (CFR 10%–19%) in humans and/or animals
8	Causes moderate morbidity and mortality (CFR 20%–29%) in humans and/or animals
10	Causes high morbidity and mortality (CFR >30%) in humans and/or animals

Note: The contribution of nematodes as plant pathogen introduction and transmission factors were recognized during the assessment. Typically, a nematode role in disease transmission is associated with the concurrent movement of infested soil on plants or equipment, infected plant material, or water runoff. The nematode itself does not possess mobility properties to move to new locations and relies upon factors that were part of the transmissibility scoring criteria. CFR- Case Fatality Rate.

**TABLE 3:** USDA plant select and non-select agents evaluated in this study.

**Select agents**	**Disease**
• *Coniothyrium glycines*	Red Leaf Blotch of Soybean
• *Peronosclerospora philippinensis (P. sacchari)*	Phillippine Downy Mildew
• *Ralstonia solanacearum phylotype II sequevar 1*	Brown Rot of Potato
• *Rathayibacter toxicus*	Annual Ryegrass Toxicity
• *Sclerophthora zeae (rayssiae)*	Brown Stripe Downy Mildew
• *Synchytrium endobioticum*	Potato Wart
• *Xanthomonas oryzae*	Bacterial blight/Leaf Streak of Rice

**Delisted or Non-Select Agent Pathogens**	**Disease**
• *Clavibacter michiganensis subsp. Nebraskensis*	Goss’ Wilt
• *Erwinia stewartii (Syn. Pantoea stewartii)*	Stewart’s Wilt
• *Magnaporthe oryzae* (*syn Pyricularia oryzae*)*Triticum pathotype*	Wheat Blast
• *Phakopsora pachyrhizi*	Asian Soybean Rust
• *Phytophthora infestans*	Late Blight of Potato
• *Puccinia graminis f.*sp. *tritici ‘Ug99’races* and variants	Wheat Stem Rust
• *Puccinia striiformis f. sp. Hordei*	Barley Stripe Rust
• *Tilletia indica (Mitra)*	*K*arnal Bunt
• Wheat Streak Mosaic Virus	Wheat Streak Mosaic Virus infection

The 9 criteria scores (1–9) were compiled for each plant pathogen and evaluated in two ways: 1) a one-dimensional (1-D) ranking where the nine scores, either unweighted or weighted (as explained below in the **Criteria weighting** section), were added for each agent, and the agents were then ranked from the lowest to the highest.; and 2) a two-dimensional (2-D) plot where the sum of the sub-scores for the “exposure” (1 + 2 + 3 + 4) branches of the hierarchy were plotted against the sum of the sub-scores for the “mitigation” (5 + 6) plus “consequences” (7 + 8 + 9) branches of the hierarchy. The 2-D plots, with unweighted and weighted sums, are shown in Figures 4, 6.

### Agent fact sheets

To challenge the assumptions behind the methodology and provide a useful test matrix, we included pathogens not currently designated as select agents but otherwise considered high risk for other purposes; these include one former select agent that has been delisted, and emerging infectious plant diseases whose potential risks are not yet fully characterized. Agent fact sheets were developed for 16 plant pathogens (Table 3) to provide the data used for scoring pathogens. Among the 16 plant pathogens are 7 current USDA plant select agents and 9 non-select plant pathogens, including two (*Magnaporthe oryzae Triticum population* and *Puccina graminis f.* sp. *tritici ‘Ug99’ races* and variants) that are non-endemic in the U.S. Due to security concerns, these fact sheets are not included as part of the manuscript but can be made available upon request to the lead author.

The agent fact sheets were created using peer-reviewed open literature sources such as Medline, PubMed, Google Scholar, and other unpublished data (data provided by SMEs) followed by thorough review by SMEs specializing in the specific pathogen. If data could not be found for a particular plant pathogen, data for similar organisms or relevant plant models was used to support scoring. In circumstances where a range of values was found (e.g., Yield loss/Marketability), the worst reasonable case (i.e., leading to the largest “bad” outcome) was typically used for scoring. In every instance, the expertise and judgment of SMEs played a crucial role in ensuring agreement on the most reliable data or foundation for scoring, especially when faced with data gaps or inconsistencies. The SMEs were asked to examine the accuracy and relevance of the information captured in the fact sheets, and the assigned scores for each data category. Any feedback received from the SMEs was integrated into the fact sheets, and adjustments to the scoring were made as necessary. SMEs providing feedback were often aware of the impact of their recommended scoring changes on the results.

**Criteria weighting.** Since not all criteria chosen for this evaluation are equivalent in terms of risk contribution, SMEs were asked to collectively assign weights (1-, 2-, or 3-fold) to the 9 criteria based on their relative importance, impact and significance to support the risk assessment. The results are shown in Table 4. Transmissibility, Host Resistance, Yield Loss/Marketability and Impact on U.S. Agriculture were given a ×3 weight; US Host Range (by crop value and economic impact), Primary Mode of Introduction, Environmental Stability, and Public/Animal Health Impact were given a 2x weight; and Countermeasures was given a 1x weight. Countermeasures do not just include chemicals but also field burning, Integrated Pest Management, quarantine, *etc.* Therefore, the countermeasures may not control or eradicate the pathogen. Even chemicals may only be effective for a limited time, i.e., developing resistance. Also, nothing is available that is able to prevent spread especially for downy mildews. Even *P. pachyrhizi* can be controlled but it has spread through the Southern U.S. soybean growing regions. Therefore, the SMEs gave it a ×1 weight.

**TABLE 4:** Proposed weight assignment by SMSs for the criteria.

	Criteria	SME assigned weight
**Exposure**	(1) U.S. host range	2
	(2) Primary Mode of introduction	2
	(3) Environmental stability	2
	(4) Transmissibility	3
**Mitigation**	(5) Countermeasures	1
	(6) Host resistance	3
**Consequence**	(7) Yield loss/marketability	3
	(8) Impact on U.S. agriculture	3
	(9) Public/animal health impact	2

Criteria and weights were combined into a single score (A) by summing all the weighted numerical values (a_i_,w_i_), where a_i_ represents a criteria score and w_i_ is the criteria weighting value:

$$A=\sum_{i=1}^{n}ai\;•\;wi$$

To facilitate comparison of results with different weighting values—the weighted case noted above and the unweighted case where all weights are assigned as 1—normalized scores were used, where the total or sub-total scores were standardized relative to a hypothetical agent that received scores of 10 in all criteria.

### Decision support framework (DSF)

**FIGURE 2:**
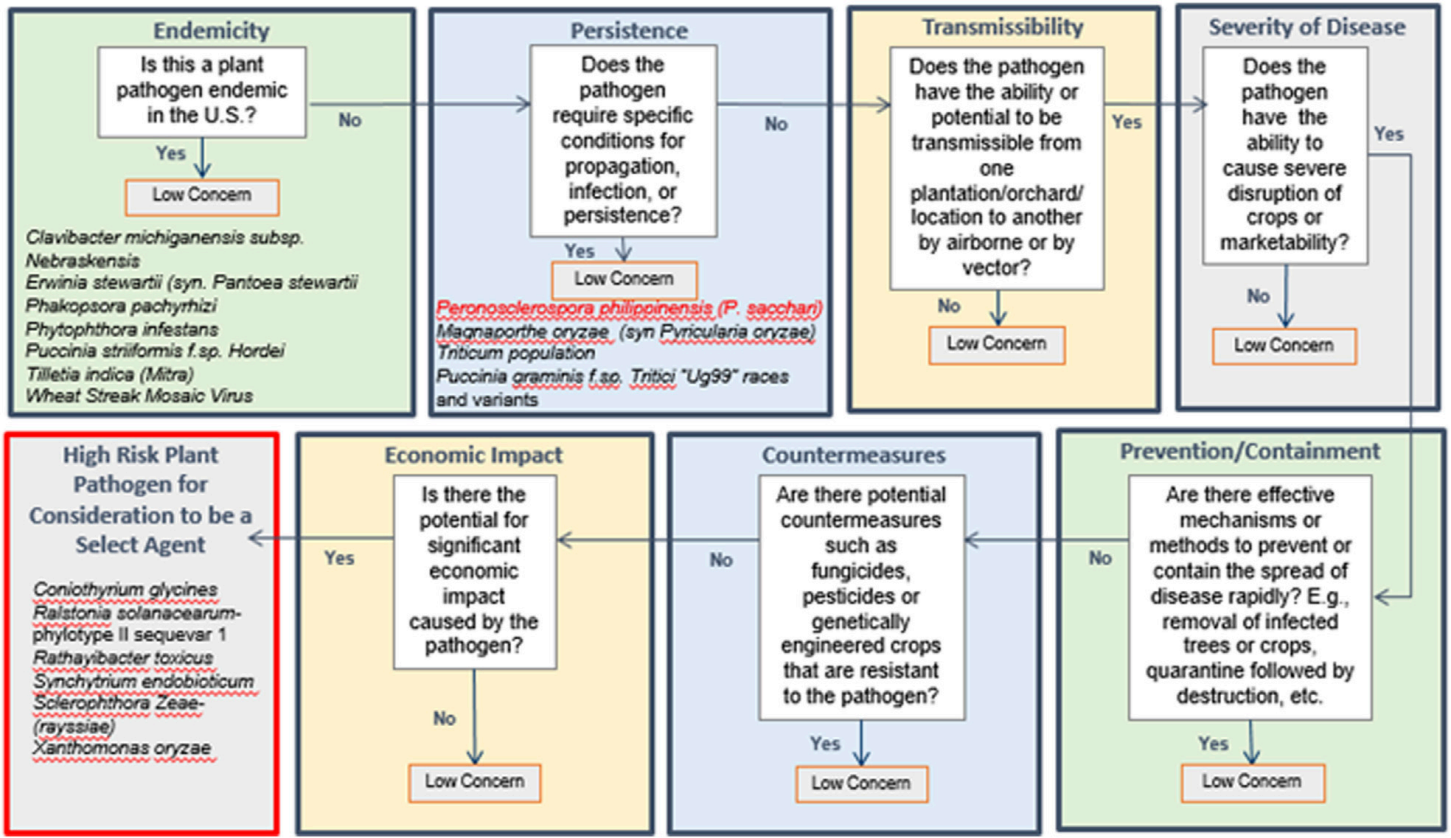
Decision Support Framework for assignments of select and non-select agents.

The DSF methodology uses a logic tree structure with key criteria to identify pathogens that may have such a low level of concern that they can be excluded from consideration as select agents (as shown in Table 5). The DSF considers both the potential impact of regulating an agent that is already present in the U.S. and the agricultural and economic consequences of a biological attack. By employing this approach, a pathogen that fails to meet the threshold value for any of the established criteria, is considered of minimal concern and is not included as a select agent. Pathogens that surpass all the threshold criteria are considered as potential select agents. Criteria encompass elements such as Endemicity, Persistence, Transmissibility, Severity of Disease, Prevention/Containment, Countermeasures, and Economic Impact. Figure 2 provides a visual representation of the DSF logic tree. Expert judgment, based on the data in the agent fact sheets, forms the basis for scoring (as indicated in Table 2). Generally, criteria receiving a score below three are indicative of a “low concern” qualitative assessment. In contrast to the MCDA approach, which employs a graded scoring system for ranking agents, the DSF approach can exclude an agent from select agent consideration based on a single criterion with a low score. Many of the criteria overlap between the MCDA and DSF approaches, except for endemicity, which is not included in the MCDA approach.

**TABLE 5:** MCDA criteria and scoring used to address questions in the DSF.

Questions	Thresholds for low concern
1. Is this plant pathogen endemic in the U.S.?^a^	Yes
2. Does this pathogen require specific conditions for propagation, infection or persistence?	Environmental Stability score of 3 or below or Disease Persistence score of 3 or below
3. Does the pathogen have the ability or potential to be transmissible from one farm, orchard, location to another by airborne or vector?	Transmissibility score of 3 or below
4. Does the pathogen have the ability to cause severe disruption of crops or marketability?	Yield Loss or Marketability Score of 3 or below
5. Are there effective mechanisms or methods to prevent or contain the spread of the disease rapidly (e.g., removal of infected trees or crops, quarantine followed by destruction, *etc.*)?	Countermeasures score of 3 or below
6. Are there potential countermeasures such as fungicides, pesticides, or genetically engineered crops that are resistant to pathogens?	Host Resistance score of 3 or below or Countermeasures score of 3 or below
7. Is there potential for significant economic impact caused by the pathogen?	Impact on U.S. Agriculture score of 3 or below

^a^ Not a MCDA, criterion.

## Results

### Unweighted ranking

As a reference point for comparison to historical Ag ISATTAC assessments where criteria were not mathematically weighted, we evaluated the unweighted (or, equivalently, equally weighted) data. To facilitate comparison of the results with current assignments as select agents and non-select agents, the two classes of agents are color coded blue and green, respectively, in the 1-D and 2-D plots.

**FIGURE 3:**
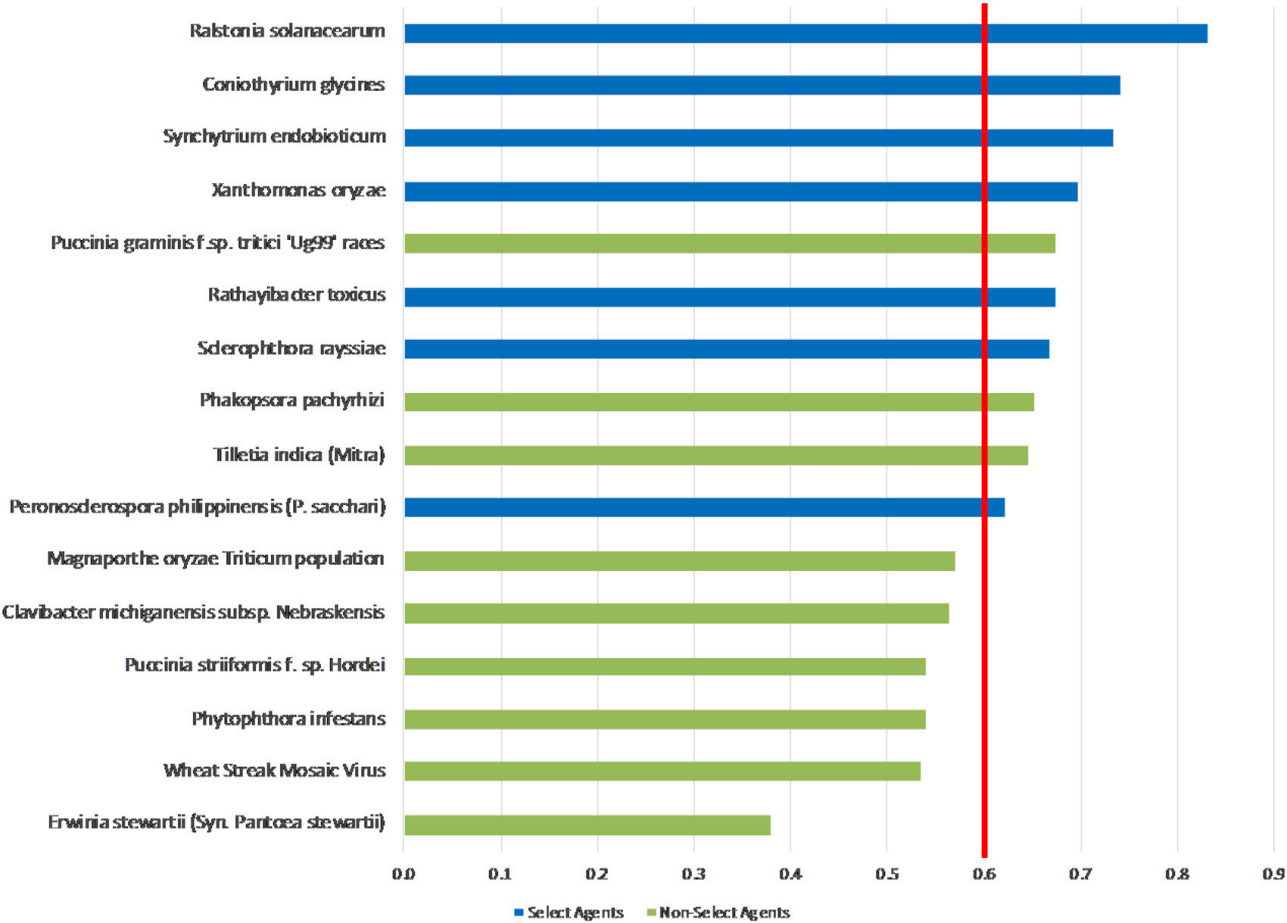
1-D plot of unweighted scoring results; select agents shown in blue and non-select agents shown in green. Score threshold, shown by red line, was chosen to be just below the lowest scoring select agent.

The 1-D unweighted results, whereby the total summated scores for all 9 pathogens are compared (Figure 3), indicated that while four select agents received the highest scores—*R*. *solanacearum, C*. *glycines, X*. *oryzae* and *S*. *endobioticum*—the other three select agents were further down in the ranking. There is currently no defined method for determining what constitutes a plant select agent. In this study, we have taken different approaches to determine if an arbitrary threshold can be established where there is a break in the data for use in evaluating future plant pathogens of concern. The proposed threshold score to distinguish high-risk from low-risk pathogens that would include the seven current select agents (e.g., score >=0.6, red line in Figure 3) would also include currently non-listed pathogens *P*. *graminis f.* sp. *tritici ‘Ug99’ races* and variants*, T*. *indica* and the delisted *P. pachyrhizi*. *Phakopsora pachyrhizi* and *P*. *graminis f.* sp. *tritici ‘Ug99’ races and variants* affect two U.S. high-value crops, soybean and wheat, respectively; have more than two modes of introduction; can be transmitted over a longer range; and could lead to large losses in yield.

**FIGURE 4:**
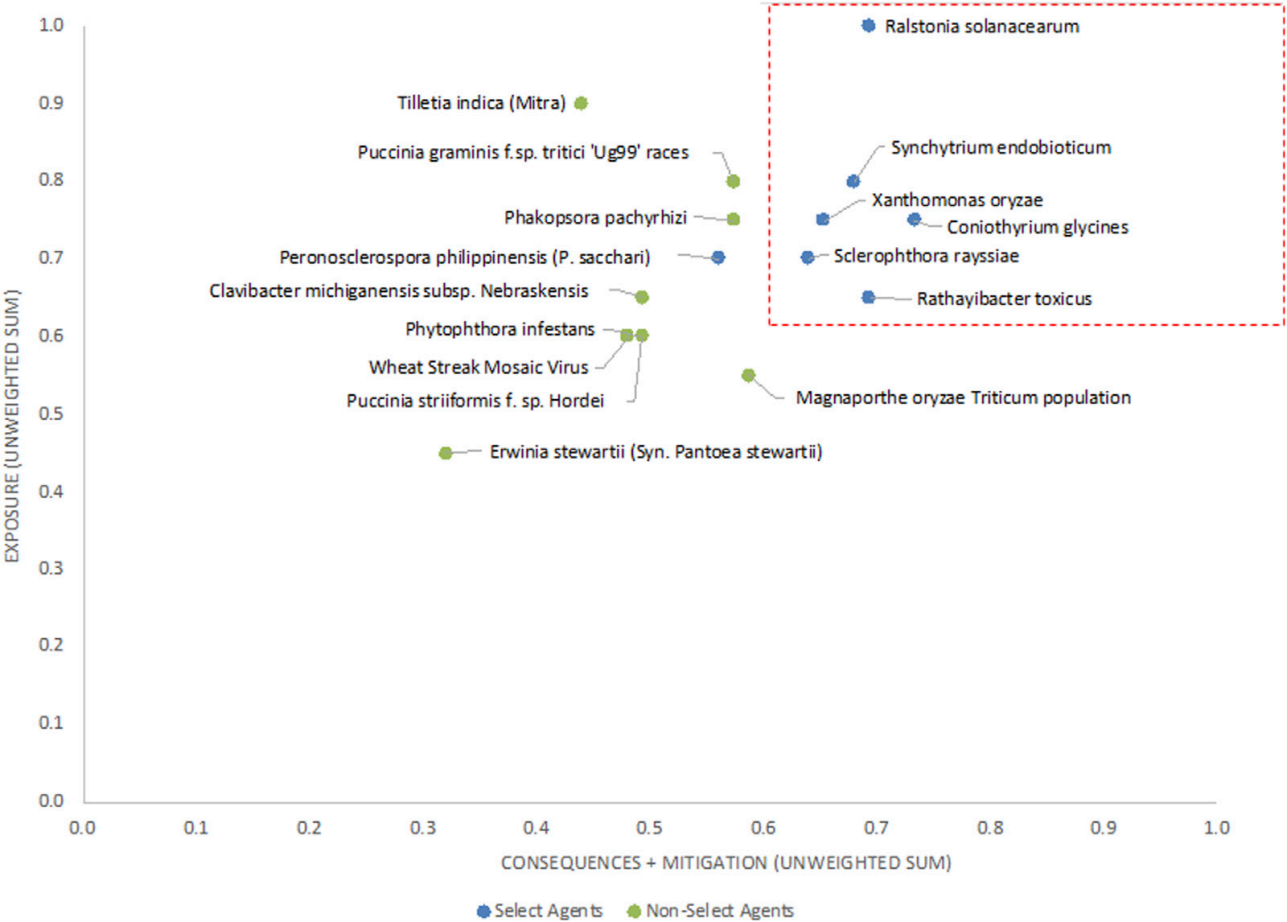
2-D plot of unweighted scoring results; select agents are shown in blue and non-select agents shown in green.

The 2-D unweighted results (Figure 4) showed similar trends, with scores for current select agents placing them generally in the upper right-hand quadrant of the plot. If thresholds based on clusters of existing select agent pathogens and breaks in data were established as scores the proposed thresholds of x >=0.60 and y>= 0.63 (red lines in Figure 4) to distinguish high-risk from low-risk pathogens, then all select agents will fall into the high-risk group except for *P*. *philippinensis* (*P. sacchari*). In this analysis, *P*. *pachyrhizi, P*. *graminis f.* sp. *tritici ‘Ug99’ races* and variants and *T*. *indica* fall outside the high-risk group. Establishing thresholds that would include *P*. *philippinensis* (*P. sacchari*) in the high-risk group (for example, adjusting the threshold to x ≥ 0.53) would also place *P*. *pachyrhizi* and *P*. *graminis f.* sp. *tritici ‘Ug99’ races* and variants in the high-risk group, while excluding *T*. *indica.*

Analysis of both the 1-D and 2-D plots indicated that, although there were general trends in the data that were consistent with current classifications, there were no sharp breaks in scoring that would serve as a basis or threshold for classifying an agent as a select agent. Instead, the plots represented a continuum of scores. Additionally, any designation of a minimal score—whether the total score in the 1-D plot, or sub-scores corresponding to the x- and y-values in the 2-D plots—resulted in some exceptions to current classifications.

### Weighted rankings

**FIGURE 5:**
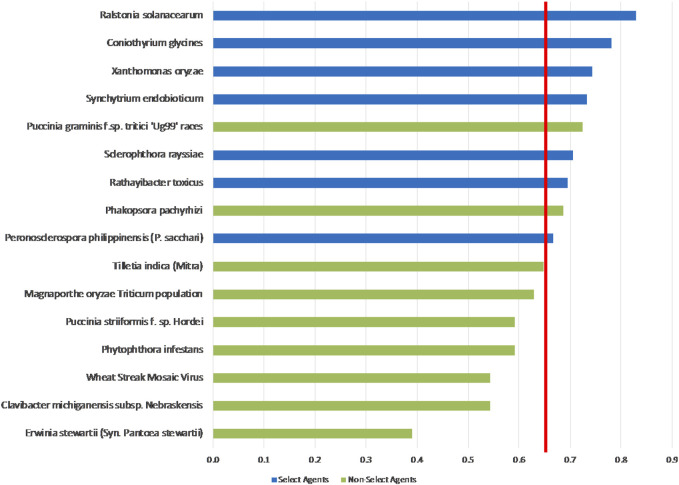
1-D plot of weighted scoring results; select agents shown in blue and non-select agents shown in green. Score threshold, shown by red line, was chosen to be just below the lowest scoring select agent.

**FIGURE 6:**
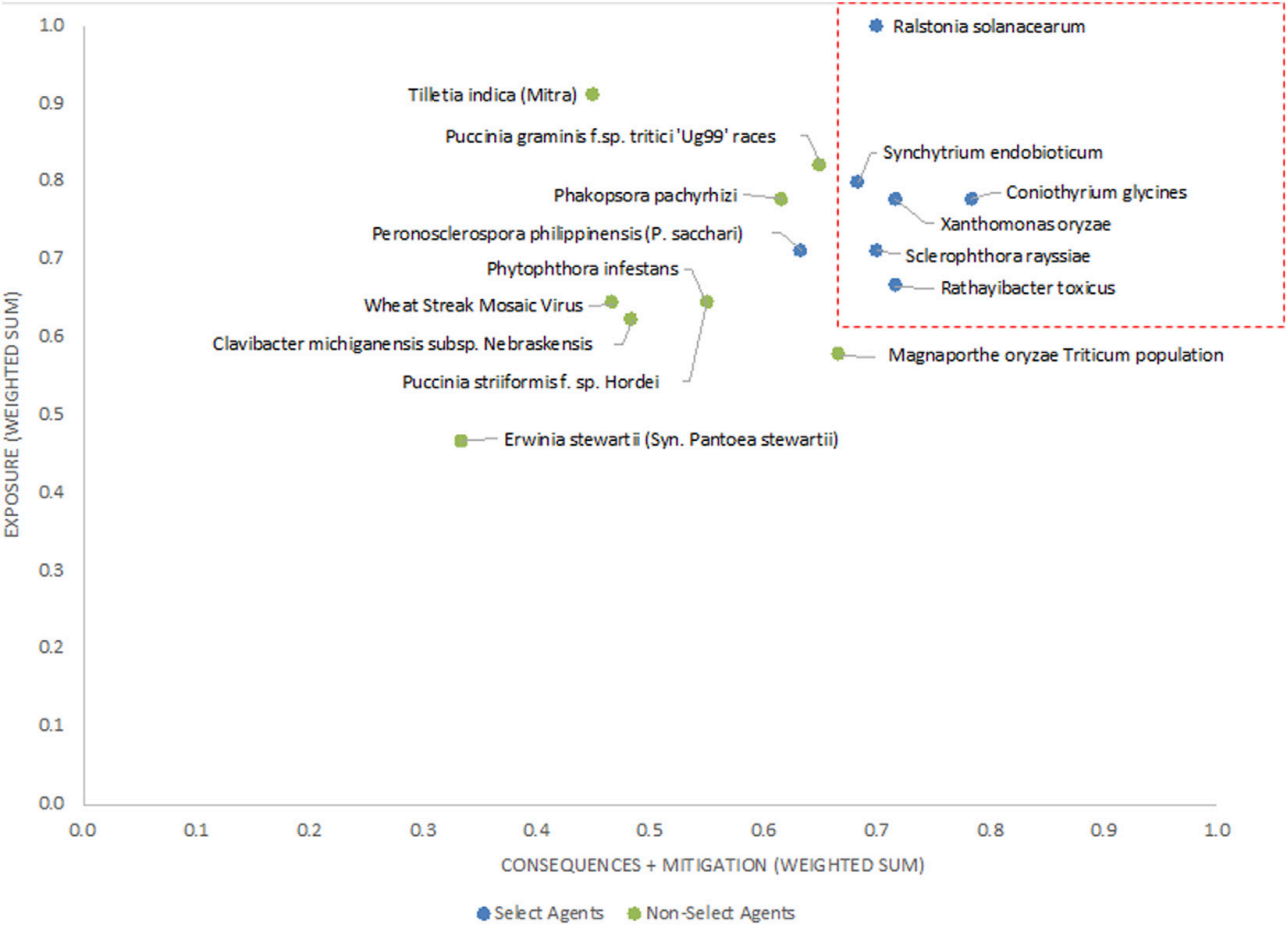
2-D plot of weighted scoring results; current select agents are shown in blue and non-select agents shown in green.

The unweighted analysis described in the previous section was repeated using the criteria weighting scheme shown in Table 4. The 1-D and 2-D plots are shown in Figures 5, 6, respectively. As observed with the unweighted data, the general trend in the data was consistent with current classifications; however, any designation of a minimal score as a basis for classification—whether the total score in the 1-D plot, or sub-scores corresponding to x- and *y*-axes values in the 2-D plots—resulted in some exceptions to current classifications. In this study, we have taken different approaches to determine if an arbitrary threshold (which may differ from the previous threshold proposed for the unweighted study) can be established where there is a break in the data for use in evaluating future plant pathogens of concern. In the 1-D ranking, four select agents received the highest scores, while two select agents (*S*. *rayssiae* and *R*. *toxicus*) ranked below *P*. *gramminis f. sp.tritici ‘Ug99’ races* and variants, and one select agent (*P*. *philippinensis (P. sacchari)*) ranked below the delisted *P*. *pachyrhizi*. In the 2-D plot, setting thresholds to distinguish high-risk and low-risk pathogens at scores x ≥ 0.675 and y ≥ 0.6 (red lines in Figure 6), all select agents scored in the high-risk group except for *P*. *philippinensis (P. sacchari)*, placing it in the lower risk grouping along with *P*. *gramminis f. sp.tritici ‘Ug99’ races* and variants, *P*. *pachyrhizi* and *T. indica*. *Peronosclerospora philippinensis (P. sacchari)*, had a low score for “Environmental Stability” and a mid-level score for “Host Resistance” which were more heavily weighted. However, the unweighted 2-D analysis also placed *P*. *philippinensis (P. sacchari)* in the low-risk group, suggesting the application of the weighting scheme shown in Table 4 did not significantly shift the relative placements enough to allow thresholds that would include *P*. *philippinensis (P. sacchari)* without also including *P*. *pachyrhizi* (previously delisted select agent) and *P*. *graminis f.* sp. *tritici ‘Ug99’ races* and variants in the high-risk group. *Phakopsora pachyrhizi* and *T. indica* are endemic to the U.S., and thus are ruled out from consideration as select agents based on this programmatic consideration.

### Decision support framework

In contrast to the MCDA approach which uses a graded scoring system for ranking agents, the DSF can rule out an agent from select agent consideration using a single low criterion score. While many of the criteria overlap between the two approaches, there are key differences such as the inclusion of “Endemicity” as the initial criterion in the DSF approach (Figure 2).

Applying the criteria for Thresholds for Low Concern listed in Table 5, seven non-select plant pathogens selected by the SMEs for inclusion in the study (which includes *T*. *indica* and previously delisted select agent *P*. *pachyrhizi*) were identified as Low Concern and removed from consideration because they are endemic in the U.S. Three additional pathogens*—P*. *philippinensis (P. sacchari), M*. *oryzae T. population* and *P*. *graminis f.* sp. *tritici ‘Ug99’races* and variants—were identified as Low Concern and removed from consideration because of the need for specific conditions for propagation, infection or poor environmental persistence. *Peronosclerospora philippinensis (P. sacchari)*, currently listed as a select agent, was removed from consideration due to poor environmental persistence, as well as the existence of available countermeasures. Based on the DSF, the following six agents were recommended for consideration to be a select agent: *C*. *glycines, R*. *solanacearum, R*. *toxicus, S*. *rayssiae, S*. *endobioticum, and X*. *oryzae.* All of these are currently listed as select agents by the USDA (Table 1 and Figure 2).

## Discussion

The overall approach employed builds on the previous Ag ISATTAC method and uses MCDA and DSF logic tree techniques. For the plant select agent tiering, we proposed initial criteria, developed fact sheets for 16 select and non-select agents using those criteria, and conducted an evaluation using the MCDA and DSF.

To our knowledge, no similar approach has been reported in the literature for assessing plant select agents across a variety of plant pathogens and U.S. crops. Criteria were selected based on 1) relevant parameters identified during the development of MCDA for human and animal health select agents, and 2) factors that addressed the statutory priorities for what constitutes a select agent for agricultural plant and plant products.

CFR Title 7, Subtitle B, Chapter 3, Part 331 currently lists the following as Plant Protection and Quarantine Select Agents based on those elements: *“Coniothyrium glycines,* (formerly *Phoma glycinicola, Pyrenochaeta glycines); Peronosclerospora philippinensis (Peronosclerospora sacchari); Ralstonia solanacearum; Rathayibacter toxicus; Sclerophthora rayssiae; Synchytrium endobioticum; and Xanthomonas oryzae”*.

The MCDA hierarchy approach breaks down the agent score into key elements of bioterrorism risk: difficulty of a successful attack (exposure), mitigation factors and consequences. While “Ease of Production” was included as a criterion for the animal and human MCDA evaluations (Pillai et al., 2022a; Pillai et al., 2023), it was weighted low and was not included here due to lack of data.

In response to plant select agent SME feedback, “Exposure” criteria were revised to better describe processes and terms specific to plants and plant pathogens. “Route of Transmission,” was updated to “Primary Mode of Introduction” to more accurately describe how a pathogen is introduced to a susceptible crop. “Aerosol stability” and “aerosol” as a mode of introduction were replaced with “Environmental Stability” which focused on the stability of the pathogen once introduced into the environment and “wind”, as aerosolized pathogens pertain to a mechanism of respiratory exposure in animals and people which does not apply to plants. The term was further refined to “Primary Mode of Introduction” to clarify that scores are based on the main mechanism that a pathogen infects a susceptible crop. Although some would argue that a vectored pathogen would pose similar risk as an aerially transmitted pathogen, it is important to note that not all vectored pathogens contribute to the same degree. Vectored pathogens that are localized (e.g., soil nematodes) *versus* pathogens that can be transmitted by flying vectors may have different transmissibility pattern and impact. “U.S. Host Range” was added as an exposure criterion to reflect the different U.S. crop species which could be impacted by a given agent.

The “Consequences” sub score was split into two categories: “Consequences” and “Mitigation,” which included “Countermeasures” and “Host Resistance.” For Host Resistance, we did not take into consideration unknown resistance simply because there is no data. For 8b, the impact associated with human health was captured under Direct Public/Animal Health Impact. Specific scoring definitions under “Countermeasures” were changed compared to human and animal criteria based on SME feedback to reflect the ways infected crop species would be addressed by industry using available chemical, physical or other measures and to incorporate whether these measures would be readily deployable.

Under “Consequences” and “Impact on U.S. Agriculture,” “Quarantine” was removed, and “Disease Persistence” added. “Quarantine” focused more on regulatory policies that may vary by jurisdiction, whereas “Disease Persistence” better described the longer-range impact on a farm. “Ability to Genetically Alter Pathogen” was removed, and “Impact on Field Production” was removed as it focused solely on field crops and was difficult to score for *X*. *oryzae* which impacts rice. “Public and Animal Health Impacts” were added to “Consequences” to include the risk of livestock mortalities from contaminated crops, such as *R*. *toxicus* toxins in livestock feed. Export impacts and endemicity were captured under “Export Trade Impacts” and “U.S. Industry Impacts”. While endemic pathogens may not be endemic across the entire U.S., SMEs agreed that those pathogens endemic anywhere in the U.S. should receive a lower score because of their existing persistence. Under “Export Trade Impacts,” endemic agents score a “2” as the criteria was updated to include “low impact due to existing control measures for endemic diseases”. Under “U.S. Industry Impacts,” endemic agents score a “0” as the criterion was updated to include low impact, as control measures are included in the overhead costs for endemic diseases.

Throughout the study, a critical element was SMEs’ contribution and feedback on the fact sheets and data interpretation. SMEs provided additional reference materials and data related to “Transmissibility” and “Primary Mode of Introduction,” resulting in a higher score for *R*. *solanacearum*, *S*. *rayssiae, P*. *graminis f.* sp. *tritici ‘Ug99’ race* and variants, and *S*. *endobioticum*, and raised questions on how to address biotrophs. SMEs also provided guidance on how to score “Disease Persistence in the U.S.” for *P*. *philippinensis (P. sacchari)*. This pathogen would most easily become persistent in weeds along the Gulf coast; however, the main crop host—corn—is not as abundant in that region. A similar concern was raised for *M. oryzae* Triticum pathotype, a pathogen which could be economically harmful, yet may be limited in spread since it favors tropical climates. Due to the high reproductive rate for *P*. *infestans* under ideal conditions, SMEs advised an increased score for “Yield Loss,” and Ag ISATTAC members recommended increasing the “Countermeasures” score for *S*. *rayssiae* to reflect the fact fungicides had a time-limited efficacy.

## Conclusion

We developed and evaluated two risk-based analytical approaches for classifying plant pathogens to support deliberations and recommendations by the Ag ISATTAC regarding which pathogens to include on the USDA Select Agent list. Previous efforts relied on SME assessments to rank the agents and did not apply the approach broadly to include non-select agent pathogens due to the additional burden placed on the SMEs. The analytical approaches presented here seek to provide a systematic approach for assessing bioterrorism risk, and to reduce the burden on SMEs by documenting the supporting data from the peer-reviewed literature in archivable data sheets. We applied the methodology broadly to evaluate the general applicability of the approach by including a variety of non-select agents in the assessment. The results of this assessment for classifying plant select agents offers a scientific and logical approach for supporting the biennial assessment of the country’s select agent programs.

Comparison of the analytical results with the current Select Agent List provided a useful reference point for evaluating these approaches and their potential impact on decision making. Both analytical approaches suggested all the current plant select agents qualify as select agents except for *P*. *philippinensis (P. sacchari)*, whereas all the non-select plant pathogens we evaluated failed to qualify as a select agent. *Puccina graminis f.* sp. *tritici ‘Ug99’ race* and variants came the closest to the thresholds for inclusion as a select agent using the MCDA method; however, it was ruled out using the DSF framework due to the need for specific conditions for propagation, infection or persistence in the environment, the same criteria that also ruled out *P. philippinensis (P. sacchari).* Climate change as an individual factor and its impact was not taken into consideration in this study in detail. However, evaluation of a pathogen’s current host range was considered. The host range can increase or decrease based upon environmental factors. *Phakopsora pachyrhizi*, a previously listed select agent, also scored close to thresholds using the MCDA approach; however, it was ruled out using the DSF framework as it is now endemic in the U.S.

Application of the methodology using both select agent and non-select agent pathogens, while helping to demonstrate the robustness of the approach, highlighted the challenges of data gaps for many pathogens and the importance of SME input and discussions. In this study the list of plant pathogens was selected collaboratively by the SMEs from USDA, other agencies and institutions to narrow our focus to refine the methodology and assess its robustness. In addition to providing risk-based tools for informing programmatic decision-making, we found that the methodologies were also useful for identifying those parameters and pathogens where more data are needed to help with prioritizing future research studies. Our future goal is to include additional pathogens as well as performing statistical and sensitivity analysis to better understand the robustness of this tool.
